# Correlation of Repeat Measurements of 27 Candidate Protein Markers for Colorectal Cancer Screening Taken Three Years and Multiple Freeze–Thaw Cycles Apart

**DOI:** 10.3390/life12030359

**Published:** 2022-03-02

**Authors:** Megha Bhardwaj, Petra Schrotz-King, Hermann Brenner

**Affiliations:** 1 Division of Clinical Epidemiology and Aging Research, German Cancer Research Center (DKFZ), 69120 Heidelberg, Germany; h.brenner@dkfz.de; 2 Division of Preventive Oncology, German Cancer Research Center (DKFZ) and National Center for Tumor Diseases (NCT), 69120 Heidelberg, Germany; petra.schrotz-king@nct-heidelberg.de; 3 German Cancer Consortium (DKTK), German Cancer Research Center (DKFZ), 69120 Heidelberg, Germany

**Keywords:** colorectal cancer, plasma proteins, sample handling, freeze–thaw cycles, proximity extension assays

## Abstract

In recent years the blood proteome has been increasingly researched for biomarkers for early detection of colorectal cancer (CRC). Blood samples from screening studies are often subject to preanalytical variability and repeated freeze–thaw cycles. We aimed to assess the correlation of repeat measurements of 27 candidate protein markers for CRC screening taken three years and multiple freeze–thaw cycles apart. The concentrations of 27 protein markers were measured in plasma samples of 39 newly detected CRC cases from a cohort of 9245 participants of screening colonoscopies. The proteins were measured using proximity extension assays (PEA) carried out on the same set of samples twice, three years apart, with an average of three freeze–thaw cycles in between the two measurements. Pearson’s product moment correlation coefficients were calculated. Correlation coefficients ranged from +0.43 to +0.97, with a median of 0.67 and an interquartile range of +0.58 to +0.84, with all *p*-values of correlation being <0.01 (<0.0005 for 22 proteins, <0.001 for 4 proteins). Repeat measurements of the 27 protein biomarkers for CRC screening performed three years later, and on average three freeze–thaw cycles apart, showed moderate to high levels of correlation. Apart from the effects of freeze–thaw cycles, slightly different preprocessing performed on the data may have contributed to recorded differences between measurements.

## 1. Introduction

In recent years the blood proteome has been increasingly researched for biomarkers for early detection of various cancers, including colorectal cancer (CRC) [1,2,3,4,5]. It is often the case in cancer screening studies for blood samples to be stored in freezers and used for multiple measurements of candidate biomarkers for early detection. If the samples are stored in bigger aliquots as a result of space constraints in large-scale screening studies, they may undergo several freeze–thaw cycles before being used for measurements of candidate biomarkers for early detection of cancer. A commonly expressed concern in this context is if, and to what extent, the measurements might suffer from freeze–thaw cycles. Although adverse effects of freeze–thaw cycles are plausible, no previous study has, to the best of our knowledge, empirically assessed their possible impact in cancer screening studies. Therefore, the objective of this study was to assess the effects of preanalytical freeze–thaw cycles on plasma proteins measured as potential biomarkers for CRC early detection among CRC cases recruited from a screening colonoscopy cohort.

## 2. Materials and Methods

### 2.1. Study Design

Blood samples were selected from participants of a screening colonoscopy collected during the BLITZ study. Details of the BLITZ study design have been reported previously [6,7,8,9,10,11]. Briefly, BLITZ is an ongoing prospective screening study of participants undergoing colonoscopies as a primary screening exam. The German screening colonoscopy program, introduced in October 2002, offers up to two screening colonoscopies at least 10 years apart to men and women aged 55 years or older [12] (starting age was lowered to 50 years for men in 2019) and includes strict measures for quality assurance. Screening colonoscopies are mostly conducted in gastroenterology practices by accredited, highly qualified and experienced endoscopists. In the BLITZ study, participants have been recruited from 20 gastroenterology practices since the end of 2005. Participants are invited to donate prediagnostic blood and stool samples and fill out self-administered questionnaires during preparatory visits at these practices typically one week before the screening colonoscopy. By the end of June 2016, out of 9425 participants in BLITZ, CRC and advanced adenoma (AA) had been detected in 64 and 633 participants, respectively. In a previous study from our group [8,9], 254 plasma samples from participants of the BLITZ study, including 41 plasma samples from participants diagnosed with CRC at the screening colonoscopy, were selected for protein measurements in the year 2015. In a later study conducted in 2018, plasma samples of partly overlapping 270 BLITZ participants, including 56 participants with CRC, were selected for another set of protein measurements [13]. Overall, there was an overlap of 39 participants with CRC and 27 protein markers included in both studies. The paired measurements in 2015 and 2018 from the same blood samples of 39 CRC patients were used for evaluating the correlation of protein measurements performed three years and three freeze–thaw cycles apart.

**Figure 1:**
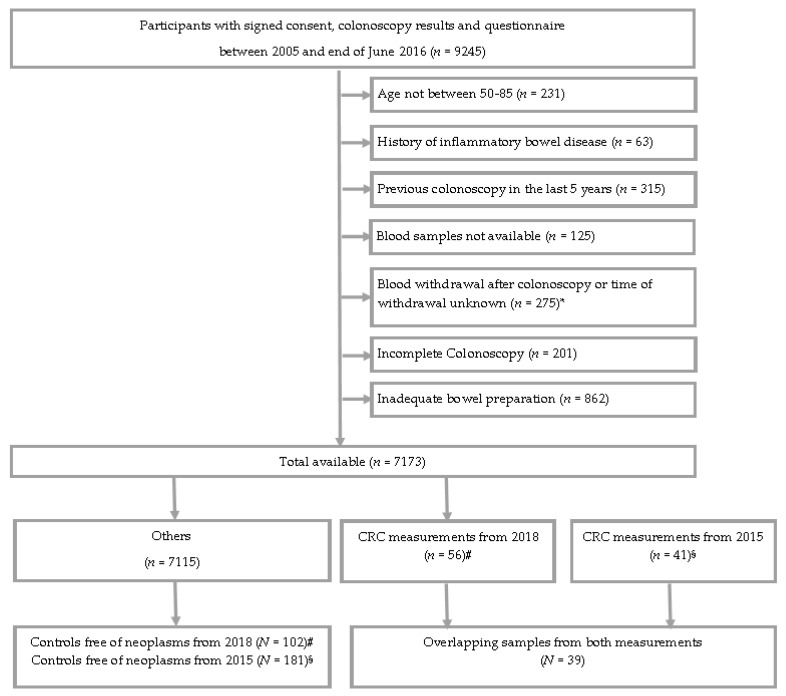
STARD (Standards for Reporting Diagnostic Accuracy Studies) flow diagram of the BLITZ study. Abbreviation: CRC, colorectal cancer. * The exclusion criteria for selection of CRC cases were not applicable after this point. # Participants selected from research study [13]. § Participants selected from research studies [8,9].

In addition to the paired measurements in the same plasma samples of 39 CRC cases, the same 27 protein markers were also measured in plasma samples from 181 and 102 control samples free of neoplasms from the 2015 and 2018 studies, respectively [8,9,13]. However, in contrast to paired-measurement cases, there was no overlap between the control samples included from the two studies. Therefore, correlations between repeat measurements that were several freeze–thaw cycles and several years apart could not be calculated for these controls. However, in order to evaluate the potential impact of freeze–thaw cycles and storing time of blood samples on diagnostic performance, we additionally assessed and compared, for each of the 27 protein markers, differences in the measurements performed among the 39 CRC cases and 102 controls in 2018, and differences in the measurements performed among the same 39 CRC cases and 181 controls in 2015. The STARD diagram showing selection of participants from the BLITZ study is presented in Figure 1 The BLITZ study has been approved by the ethics committees of the Medical Faculty of University of Heidelberg (S-178/2005), and of the physicians’ boards of Baden-Wuerttemberg (M118-05-f), Rhineland-Palatinate (837.047.06(5145)) and Saarland (217/13). The BLITZ study adheres to the standards set by the Declaration of Helsinki, and all study participants voluntarily provided written informed consent.

### 2.2. Sample Collection and Storage and Lab Assay

The blood draw was performed prior to first diagnosis at recruitment in the BLITZ study. After blood draw, Ethylenediaminetetraacetic acid (EDTA) plasma samples were transported to the laboratory while preserved in a cold transport chain, followed by centrifugation at 2000–2500× *g* for 10 min at 4 °C and then stored at −80 °C until picked out for the protein measurements.

**Figure 2:**
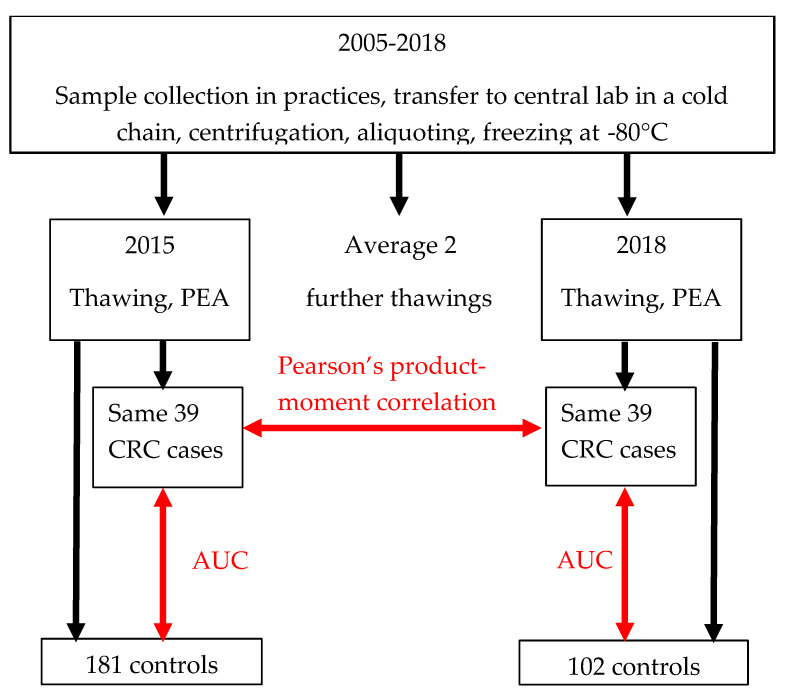
Study workflow. Abbreviations: AUC, area under the receiver operating curve; CRC, colorectal cancer; PEA, proximity extension assay.

Protein concentrations in plasma samples were measured utilizing the proximity extension assay (PEA) offered by Olink. Olink’s multiplex panels allow simultaneous analysis of 92 biomarkers and 4 internal controls in samples of 1 µL [14]. Briefly, the 96 pairs of oligonucleotide-labelled antibodies are allowed to pairwise bind to target proteins; when in close proximity, a PCR reporter sequence is formed as a result of DNA polymerization, which is quantified by real-time PCR. The first measurement was performed in 2015 for 92 proteins that were measured using Olink’s Proseek^®^ Multiplex Oncology I panel [9] in plasma samples of 41 CRC cases. Subsequently, in the year 2018, 92 proteins were measured using Olink’s Proseek^®^ Multiplex Oncology II panel in plasma samples of 56 CRC cases [13]. In total, there was an overlap of 27 proteins between the two Olink panels for the 39 CRC cases that were included in both studies. There was an average difference of three years and three freeze–thaw cycles between the two measurements. In addition to the paired measurements for the 39 CRC cases, the 27 proteins were also measured in 181 and 102 non-overlapping control samples free of neoplasms from the years 2015 and 2018, respectively. Blind laboratory analyses were performed with respect to findings at colonoscopy in the laboratory of the manufacturer of the panels. The workflow of the current study is presented in Figure 2.

### 2.3. Statistical Analyses

Normalization was performed in order to minimize both inter- and intra-assay variation. Nevertheless, there were minor differences in the preprocessing of the data. The x-value of the point in qPCR where the reaction curve intersects with the threshold line is called the Cq value. Normalization in the first measurement in 2015 was a two-step procedure, with the first step being to subtract the raw Cq value from the extension control in order to correct for technical variation. The second step was to further normalize the calculated dCq-value against the negative control determined in the measurement, which yielded ddCq values (hereafter referred to as Cq values) on a log2 scale [8].

However, for the second measurement, normalization was a three-step procedure to obtain an arbitrary, relative quantification unit called Normalized Protein Expression (NPX). NPX is derived from the Ct values obtained from the qPCR by first subtracting the raw Ct value of an analyte from the Ct of extension controls. The obtained dCt of an analyte is further subtracted from dCt of interplate control in order to obtain the ddCt of an analyte. In the final step, the predetermined correction factor is subtracted from the ddCt of the analyte in order to obtain the NPX of any protein. NPX is an arbitrary unit and represents the relative signal on a log2 scale; a one-unit NPX difference represents a two-fold difference in protein concentration.

For assessing concordance between the first measurements from 2015 and the second measurements from 2018 of the 27 proteins included in both measurements, the Pearson’s product-moment correlation coefficient was calculated for the 39 CRC cases with paired measurements. In addition, the means, standard deviations and standard errors for the first and second measurements of the 27 protein biomarkers were calculated.

Complementary to direct comparisons of the paired 39 CRC samples were assessed and compared for each of the 27 protein markers; differences in the measurements performed among the 39 CRC cases and 102 controls in 2018, as well as differences in the measurements performed among the same 39 CRC cases and 181 controls in 2015 were also assessed and compared. Differences between cases and controls for each of the 27 proteins were assessed for statistical significance using the Wilcoxon rank-sum test with adjustments made for multiple testing using the Benjamini–Hochberg method [15]. Furthermore, for each individual protein biomarker, a logistic regression model was used in order to construct the prediction algorithm for the presence of advanced neoplasms, while predictive performance was assessed using AUCs and their 95% confidence intervals (95% CI). Correlation across the 27 proteins of the AUCs between the 2015 and 2018 measurements was quantified by Pearson’s correlation coefficient, and the DeLong test was used to determine the statistical significance of differences between AUCs for each protein from the 2015 and 2018 measurements [16]. All statistical analyses were performed with the R statistical software language and environment (version 3.5.0, R core team) [17], and *p*-values < 0.05 in two-sided testing were considered to be statistically significant.

## 3. Results

### 3.1. Characteristics of the Study Population

The eligibility criteria used for selection of study participants enrolled and selected from the *Begleitende Evaluierung innovativer Testverfahren zur Darmkrebs-Früherkennung* (BLITZ) study are presented in Figure 1 displaying the Standards for Reporting Diagnostic Accuracy studies (STARD) diagram. The main characteristics of the study population are presented in Table 1.

The sample of 39 CRC patients for whom the same 27 proteins were measured twice (with an average difference of three freeze–thaw cycles) from the same plasma samples in 2015 and 2018 included 28 men and 11 women, with a median age of 67 years. CRC was detected at stages I–III in 36 out of 39 cases. Controls whose samples were measured in 2015 and 2018 were on average slightly younger with median ages of 62 and 65.5 years, respectively.

For all protein biomarkers the intra- and inter-coefficients of variation were <20% for all measurements. The limit of detection was set three standard deviations above the background for protein. The internal and external controls developed by Olink enable monitoring of assay performance and quality of samples, and detection control monitors the read out. Extension control facilitates normalization across samples and inter-plate control enables normalization between plates.

### 3.2. Correlation Analyses

**Figure 3:**
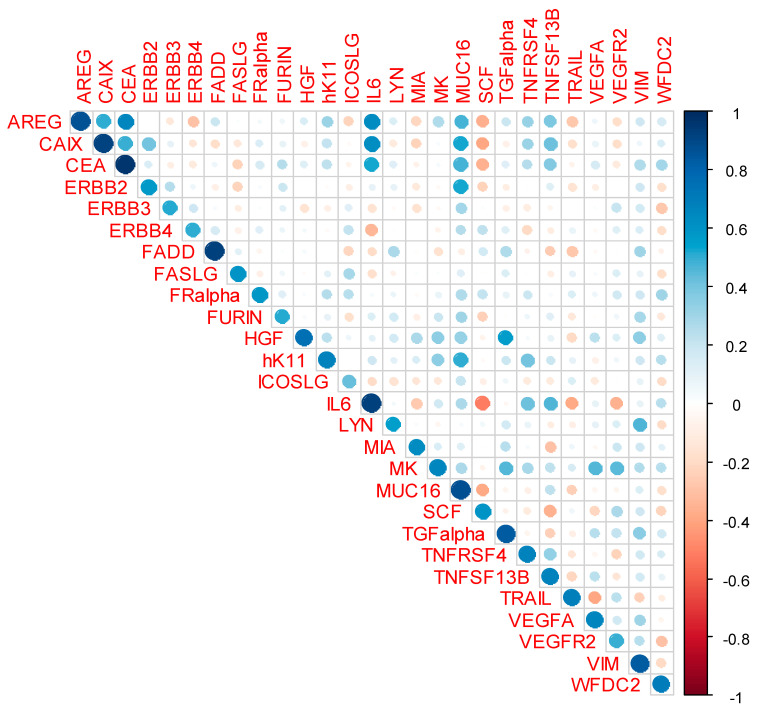
Correlation plot displaying Pearson’s product–moment correlation of the 27 markers from the measurements of 2015 and 2018. Abbreviations: AREG, amphiregulin; CAIX, carbonic anhydrase IX; CEA, carcinoembryonic antigen; ERBB2, receptor tyrosine-protein kinase erbB-2; ERBB3, receptor tyrosine-protein kinase erbB-3; ERBB4, receptor tyrosine-protein kinase erbB-4; FADD, Fas-associated death domain protein; FASLG, Fas antigen ligand; FRalpha, folate receptor alpha; HGF, hepatocyte growth factor; hK11, kallikrein 11; ICOSLG, ICOS ligand; IL6, interleukin-6; LYN, tyrosine-protein kinase Lyn; MIA, melanoma-derived growth regulatory protein; MK, midkine; MUC16, mucin-16; NPX, normalized protein expression; SCF, stem cell factor; TGFalpha, transforming growth factor alpha; TNFRSF4, tumor necrosis factor receptor superfamily member 4; TNFSF13B, tumor necrosis factor ligand superfamily member 13B; TRAIL, TNF-related apoptosis-inducing ligand; VEGFA, vascular endothelial growth factor A; VEGFR2, vascular endothelial growth factor receptor 2; VIM, vimentin; WFDC2, WAP four-disulfide core domain protein 2.

**Figure 4:**
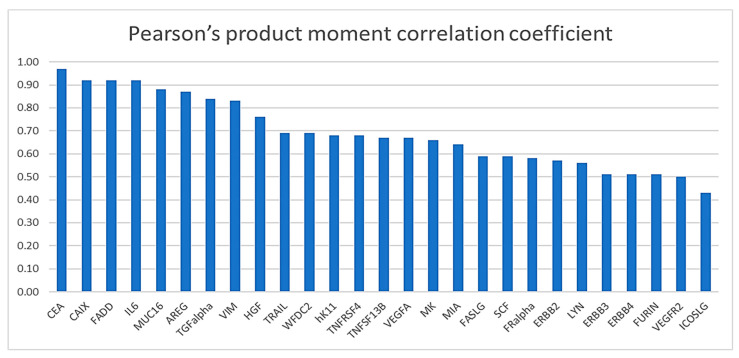
Distribution of Pearson’s product–moment correlation coefficients of the markers from the measurements of 2015 and 2018. Abbreviations: AREG, amphiregulin; CAIX, carbonic anhydrase IX; CEA, carcinoembryonic antigen; ERBB2, receptor tyrosine-protein kinase erbB-2; ERBB3, receptor tyrosine-protein kinase erbB-3; ERBB4, receptor tyrosine-protein kinase erbB-4; FADD, Fas-associated death domain protein; FASLG, Fas antigen ligand; FRalpha, folate receptor alpha; HGF, hepatocyte growth factor; hK11, kallikrein 11; ICOSLG, ICOS ligand; IL6, interleukin-6; LYN, tyrosine-protein kinase Lyn; MIA, melanoma-derived growth regulatory protein; MK, midkine; MUC16, mucin-16; NPX, normalized protein expression; SCF, stem cell factor; TGFalpha, transforming growth factor alpha; TNFRSF4, tumor necrosis factor receptor superfamily member 4; TNFSF13B, tumor necrosis factor ligand superfamily member 13B; TRAIL, TNF-related apoptosis-inducing ligand; VEGFA, vascular endothelial growth factor A; VEGFR2, vascular endothelial growth factor receptor 2; VIM, vimentin; WFDC2, WAP four-disulfide core domain protein 2.

The results of correlation analyses of the repeat measurements (2015 and 2018) of the 27 proteins in the same plasma samples from the 39 CRC cases are presented in Table 2 and in Figure 3 and Figure 4. With correlation coefficients ranging from +0.43 to +0.97 (median +0.67, interquartile range +0.58 to +0.84), moderate to high positive correlations were observed between the first and second measurements for all 27 proteins (*p*-values < 0.01 for all proteins and <0.001 for 26 out of 27 proteins). With correlation coefficients between +0.86 and +0.97, correlations were very high for these six proteins: amphiregulin (AREG), carbonic anhydrase (CAIX), carcinoembryonic antigen (CEA), Fas-associated death domain protein (FADD), interleukin-6 (IL6) and mucin-16 (MUC16). Strong correlations with correlation coefficients between +0.66 and +0.84 were still observed for the following ten proteins: hepatocyte growth factor (HGF), kallikrein 11 (hk11), midkine (MK), transforming growth factor alpha (TGFalpha), tumor necrosis factor receptor superfamily member 4 (tumor necrosis factor receptor superfamily member 4 (TNFRSF4), tumor necrosis factor ligand superfamily member 13B (TNFSF13B), TNF-related apoptosis-inducing ligand (TRAIL), vimentin (VIM), vascular endothelial growth factor A (VEGFA) and WAP four-disulfide core domain protein 2 (WFDC2).

The mean concentration Cq and NPX values of the first and second measurements, respectively, for all the proteins are also presented in Table 2. As the normalizations performed on datasets from both the measurements were slightly different, the absolute concentration values are not directly comparable. Nevertheless, the standard deviations and standard errors of both measurements were largely similar.

### 3.3. Analyses of Diagnostic Performance

Results of the univariate analysis comparing the expression difference of each marker in plasma samples from 39 CRC cases to 181 controls from 2015 and the same 39 CRC cases to 102 controls from the measurements in 2018 are reported in Table 3. Statistically significant expression differences between CRC cases and controls without neoplasms were found for six proteins, and persisted for five proteins after correction for multiple testing in the 2015 measurements. Two proteins, AREG and CEA, displayed areas under the ROC curves (AUCs) ≥0.7 for discrimination of CRC cases from controls. Similarly, for the measurements from 2018 that compared 39 CRC cases to 102 controls free of neoplasms, adjusted *p*-values ≤0.05 were observed for six protein biomarkers and AUCs ≥0.7 were observed for the same two biomarkers, AREG and CEA, in the second measurements. The AUCs for the 2015 and 2018 measurements were positively correlated, with a Pearson correlation coefficient of +0.58; for only 1 out of the 27 proteins (WFDC2), a statistically significant difference in AUC was seen between the 2015 and 2018 measurements.

## 4. Discussion

In order to determine the effect of freeze–thaw cycles and prolonged sample storage times on expressions of candidate plasma proteins for CRC early detection, concentrations of 27 proteins were directly compared for paired samples from proximity extension assays (PEA) measurements performed in the year 2015 and, after an additional three freeze–thaw cycles, in 2018. Paired measurements were available for 39 CRC cases detected at screening colonoscopy. Moderate to very strong correlations were seen for all 27 proteins, with correlation coefficients ranging from 0.43 to 0.97 (*p* < 0.001 for 26 out of 27 proteins). Furthermore, analyses of diagnostic performance based on samples from the two different measurement rounds yielded rather similar results, despite inclusion of different samples of controls.

Large-scale screening and cohort studies are an invaluable resource for evaluating novel biomarkers for cancer screening. However, they often require many years of participant recruitment and follow-up. For reasons related to efficiency and logistics, and to minimize batch effects, laboratory analyses are typically performed in single batches after completion of recruitment and/or follow-up, and the precious biospecimens collected in such studies are typically stored frozen for long times at very low temperatures in order to remain available for additional analyses of novel emerging biomarkers; or to use novel, emerging technologies upon. As a result of limited storage capacities for very small aliquots, larger blood aliquots must sometimes be used multiple times and samples consequently undergo multiple freeze–thaw cycles before analysis. The research of protein biomarkers is mired by several factors, such as the dynamic range of plasma proteins [18], inconsistencies in sample handling, preanalytical and analytical procedures [19,20], in addition to varying times required for sample fixation after sample processing [21]. Although there is consensus that storing times might be of concern for some markers [22,23] and that freeze–thaw cycles should be minimized wherever possible, there is very limited empirical evidence pertaining to the degree to which prolonged storing times and freeze–thaw cycles compromise the reliability and validity of measurements of candidate diagnostic markers; moreover, there are few estimates of their diagnostic performance.

Our study aimed to help fill in these gaps for plasma measurements of 27 candidate diagnostic proteins performed in two batches, three years apart. Overall, rather high correlations were seen between repeat measurements, despite the large differences in sample handling in terms of storage times and freeze–thaw cycles. It is worth noting in this context that even repeat measurements of the same samples without any additional storage time or freeze–thaw cycle would not be expected to yield perfect correlations; variations between the two measurements may not be fully ascribed to sample storage times and freeze–thaw cycles. Although our results are reassuring in that measurement correlations for the majority of the candidate protein markers were very high, they also illustrate that vulnerabilities to preanalytical handling may strongly vary between proteins. Therefore, neither general disregard for samples that have been stored for long time and undergone multiple freeze–thaw cycles, nor general ignorance of the relevance of preanalytical conditions, are warranted. Rather, a differentiated view regarding specific analytical conditions and their relevance for specific biomarkers is warranted. In this respect, our study aims to contribute to the very limited empirical evidence available thus far.

Our study has specific strengths and limitations. A major strength is its conduction in a true screening setting, and in the target population of CRC screening for whom potential inclusion of the candidate proteins in a blood-based screening test would be of immediate practical relevance. Many studies on the role of pre-analytics for diagnostic measurements have relied on small convenience samples. In our study, the presence of CRC and absence of colorectal neoplasms were confirmed by screening colonoscopy in all participants. The study was based on a very large cohort of screening colonoscopy participants that enabled inclusion of a reasonably sized sample of screening-detected CRC patients, despite the very low prevalence of CRC in the screening population. In addition to providing quantitative evidence for the correlation between plasma protein measurements, our study also provides empirical evidence for the potential impact of preanalytical history on estimates of diagnostic performance. However, a number of limitations also need to be addressed. Although the same proteins were measured in both rounds of measurements, they were included in different protein panels in the first and second measurements, which may have affected measurement quality. Furthermore, different types of normalization were employed in the first and second measurement rounds. Finally, direct comparisons of measurements were restricted to screened subjects in whom CRC was detected at colonoscopy, whereas no such direct comparisons were possible among control samples due to a lack of overlap among them.

Despite these limitations, our results provide valuable empirical evidence on the stability against major differences in preanalytics for a number of plasma proteins that may be relevant for a potential CRC multi-protein screening panel. In particular, very high correlations were found for several markers showing the highest diagnostic potential in this and previous analyses, such as AREG and CEA [8,9,13,24], but wide variations in correlation were seen across various proteins. Although biomarkers such as AREG and CEA are not ready for clinical use, using a combination of these potentially promising proteins with other types of genetic, epigenetic, protein or metabolomic biomarkers could contribute to the development of a powerful blood-based screening tool. Such multi-omics development may receive further momentum through the application of artificial intelligence tools [25,26]. Given that preanalytics may not often be perfect in routine screening practices, the robustness of markers against various preanalytical conditions is an additional, important criterion to be considered in biomarker research that should receive increased attention in future studies.

## Figures and Tables

**Table 1:** Main characteristics of the study population.

Characteristic	CRC Cases with Repeat Measurements from Same Plasma Samples in 2015 and 2018		Controls Measured in 2015		Controls Measured in 2018	
	N (39)	%	N (181)	%	N (102)	%
**Age in Years**						
50–59	7	18	67	37	21	21
60–69	20	51	81	45	50	49
70–84	12	31	33	18	31	30
Mean	66.5	-	62.9	-	65.4	-
Median	67.0	-	62.0	-	65.5	-
SD	6.6	-	6.2	-	6.9	-
**Gender Distribution**						
Male	28	72	103	57	66	65
Female	11	28	78	43	36	35
**Stage Distribution**						
Stage I	13	33	-	-	-	-
Stage II	3	8	-	-	-	-
Stage III	20	51	-	-	-	-
Stage IV	3	8	-	-	-	-

Abbreviations: CRC, colorectal cancer; N, number; SD, standard deviation.

**Table 2:** Correlation coefficients, mean concentrations, standard deviations and standard errors of repeat measurements of 27 proteins from the same plasma samples of 39 CRC cases analysed in 2015 and 2018.

Marker	Pearson’s Product-Moment Correlation Coefficient	*p*-Value *	Measurements from 2015			Measurements from 2018		
			Mean Cq	Standard Deviation	Standard Error	Mean NPX	Standard Deviation	Standard Error
CEA	0.97	<0.0005	3.70	1.43	0.23	2.17	1.45	0.23
CAIX	0.92	<0.0005	2.87	1.29	0.21	3.63	1.10	0.18
FADD	0.92	<0.0005	3.95	1.15	0.18	2.52	1.06	0.17
IL6	0.92	<0.0005	4.99	0.88	0.14	3.02	0.85	0.14
MUC16	0.88	<0.0005	2.71	1.05	0.17	4.05	0.87	0.14
AREG	0.87	<0.0005	3.50	0.65	0.10	2.64	0.59	0.10
TGFalpha	0.84	<0.0005	4.43	0.83	0.13	4.34	0.83	0.13
VIM	0.83	<0.0005	5.85	0.81	0.13	5.88	0.69	0.11
HGF	0.76	<0.0005	8.35	0.50	0.08	8.51	0.57	0.09
TRAIL	0.69	<0.0005	7.90	0.28	0.04	7.77	0.35	0.06
WFDC2	0.69	<0.0005	7.55	0.42	0.07	8.17	0.46	0.07
hK11	0.68	<0.0005	5.72	0.51	0.08	6.33	0.50	0.08
TNFRSF4	0.68	<0.0005	3.50	0.48	0.08	3.13	0.43	0.07
TNFSF13B	0.67	<0.0005	6.68	0.35	0.06	6.93	0.35	0.06
VEGFA	0.67	<0.0005	10.89	0.47	0.08	10.04	0.58	0.09
MK	0.66	<0.0005	7.74	0.60	0.10	6.95	0.68	0.11
MIA	0.64	<0.0005	4.97	0.34	0.05	10.21	0.31	0.05
FASLG	0.59	<0.0005	2.40	0.42	0.07	9.09	0.44	0.07
SCF	0.59	<0.0005	8.25	0.49	0.08	9.09	0.53	0.08
FRalpha	0.58	<0.0005	4.89	0.46	0.07	6.38	0.49	0.08
ERBB2	0.57	<0.0005	8.95	0.44	0.07	7.89	0.36	0.06
LYN	0.56	<0.0005	2.77	0.74	0.12	3.55	0.87	0.14
ERBB3	0.51	<0.001	8.94	0.38	0.06	7.92	0.26	0.04
ERBB4	0.51	<0.001	5.83	0.38	0.06	5.18	0.33	0.05
FURIN	0.51	<0.001	7.74	0.38	0.06	9.34	0.38	0.06
VEGFR2	0.50	<0.001	7.88	0.43	0.07	7.17	0.28	0.04
ICOSLG	0.43	<0.01	4.03	0.38	0.06	5.14	0.39	0.06

* *p*-value is the level of significance of the t-test from Pearson’s product–moment correlation that indicates whether variables are significantly correlated or not. Abbreviations: AREG, amphiregulin; CAIX, carbonic anhydrase IX; CEA, carcinoembryonic antigen; ERBB2, receptor tyrosine-protein kinase erbB-2; ERBB3, receptor tyrosine-protein kinase erbB-3; ERBB4, receptor tyrosine-protein kinase erbB-4; FADD, Fas-associated death domain protein; FASLG, Fas antigen ligand; FRalpha, folate receptor alpha; HGF, hepatocyte growth factor; hK11, kallikrein 11; ICOSLG, ICOS ligand; IL6, interleukin-6; LYN, tyrosine-protein kinase Lyn; MIA, melanoma-derived growth regulatory protein; MK, midkine; MUC16, mucin-16; NPX, normalized protein expression; SCF, stem cell factor; TGFalpha, transforming growth factor alpha; TNFRSF4, tumor necrosis factor receptor superfamily member 4; TNFSF13B, tumor necrosis factor ligand superfamily member 13B; TRAIL, TNF-related apoptosis-inducing ligand; VEGFA, vascular endothelial growth factor A; VEGFR2, vascular endothelial growth factor receptor 2; VIM, vimentin; WFDC2, WAP four-disulfide core domain protein 2.

**Table 3:** Diagnostic performance of individual markers for detecting CRC at two different time points with an average of three freeze–thaw cycles.

Marker	Measurements from 2015 (nCRC = 39, nControls = 181)					Measurements from 2018 (nCRC = 39, nControls = 102)					DeLong *p*-val for Testing Differences in AUCs
	Mean Cq		AUC (95% CI)	*p*-val	*p*-val^adj^	Mean NPX		AUC (95% CI)	*p*-val	*p*-val^adj^	
	CRC	Controls				CRC	Controls				
AREG	3.50	2.95	0.79 (0.71–0.86)	<0.05	<0.05	2.64	2.27	0.71 (0.61–0.80)	<0.05	<0.05	0.20
CAIX	2.87	2.62	0.54 (0.44–0.64)	0.46	0.72	3.63	3.50	0.51 (0.40–0.62)	0.91	0.91	0.68
CEA	3.70	2.65	0.75 (0.66–0.84)	<0.05	<0.05	2.17	1.30	0.70 (0.60–0.81)	<0.05	<0.05	0.52
ERBB2	8.95	8.85	0.57 (0.47–0.68)	0.15	0.36	7.89	7.92	0.52 (0.41–0.63)	0.76	0.85	0.47
ERBB3	8.94	8.91	0.53 (0.42–0.63)	0.62	0.72	7.92	7.95	0.54 (0.43–0.65)	0.49	0.60	0.87
ERBB4	5.83	5.79	0.53 (0.43–0.63)	0.58	0.72	5.18	5.24	0.56 (0.46–0.67)	0.24	0.43	0.64
FADD	3.95	3.91	0.50 (0.40–0.60)	0.98	0.98	2.52	2.19	0.60 (0.50–0.70)	0.07	0.21	0.17
FASLG	2.40	2.45	0.47 (0.38–0.57)	0.60	0.72	9.09	9.10	0.51 (0.40–0.62)	0.87	0.91	0.62
FRalpha	4.89	4.95	0.53 (0.43–0.63)	0.53	0.72	6.38	6.57	0.64 (0.53–0.75)	<0.05	<0.05	0.15
FURIN	7.74	7.68	0.52 (0.42–0.62)	0.66	0.74	9.34	9.27	0.54 (0.44–0.65)	0.41	0.56	0.76
HGF	8.35	8.03	0.65 (0.56–0.74)	<0.05	<0.05	8.51	8.31	0.60 (0.50–0.71)	0.05	0.21	0.52
hK11	5.72	5.61	0.56 (0.46–0.67)	0.21	0.42	6.33	6.43	0.59 (0.48–0.70)	0.11	0.25	0.76
ICOSLG	4.03	4.01	0.53 (0.43–0.63)	0.55	0.72	5.14	5.32	0.65 (0.54–0.75)	<0.05	<0.05	0.12
IL6	4.99	4.63	0.64 (0.55–0.72)	<0.05	<0.05	3.02	2.87	0.56 (0.46–0.66)	0.27	0.43	0.25
LYN	2.77	2.96	0.57 (0.49–0.66)	0.15	0.36	3.55	3.23	0.60 (0.50–0.70)	0.07	0.21	0.70
MIA	4.97	4.93	0.54 (0.45–0.63)	0.43	0.72	10.21	10.26	0.56 (0.45–0.67)	0.27	0.45	0.78
MK	7.74	7.74	0.51 (0.41–0.61)	0.82	0.88	6.95	6.80	0.57 (0.46–0.67)	0.22	0.43	0.46
MUC16	2.71	2.38	0.60 (0.49–0.71)	0.05	0.16	4.05	3.87	0.55 (0.45–0.66)	0.32	0.48	0.55
SCF	8.25	8.24	0.50 (0.40–0.61)	0.98	0.98	9.09	9.14	0.55 (0.44–0.65)	0.37	0.52	0.53
TGFalpha	4.43	4.06	0.62 (0.53–0.72)	<0.05	0.07	4.34	3.97	0.64 (0.54–0.75)	<0.05	<0.05	0.80
TNFRSF4	3.50	3.44	0.53 (0.43–0.62)	0.59	0.72	3.13	3.20	0.57 (0.46–0.67)	0.22	0.43	0.59
TNFSF13B	6.68	6.61	0.55 (0.45–0.65)	0.33	0.62	6.93	6.87	0.54 (0.44–0.65)	0.45	0.57	0.91
TRAIL	7.90	7.96	0.57 (0.47–0.67)	0.18	0.38	7.77	7.97	0.67 (0.57–0.77)	<0.05	<0.05	0.17
VEGFA	10.89	10.69	0.60 (0.51–0.68)	0.05	0.17	10.04	9.88	0.59 (0.49–0.69)	0.11	0.25	0.87
VEGFR2	7.88	7.81	0.46 (0.36–0.56)	0.45	0.72	7.17	7.21	0.48 (0.37–0.59)	0.72	0.85	0.80
VIM	5.85	5.47	0.59 (0.50–0.68)	0.07	0.20	5.88	5.59	0.59 (0.48–0.69)	0.10	0.25	0.96
WFDC2	7.55	7.31	0.64 (0.55–0.74)	<0.05	<0.05	8.17	8.14	0.49 (0.39–0.60)	0.87	0.91	0.03

Abbreviations: AREG, amphiregulin; AUC, area under the receiver operating curve; CAIX, carbonic anhydrase IX; CEA, carcinoembryonic antigen; CRC, colorectal cancer; ERBB2, receptor tyrosine-protein kinase erbB-2; ERBB3, receptor tyrosine-protein kinase erbB-3; ERBB4, receptor tyrosine-protein kinase erbB-4; FADD, Fas-associated death domain protein; FASLG, Fas antigen ligand; FRalpha, folate receptor alpha; HGF, hepatocyte growth factor; hK11, kallikrein 11; ICOSLG, ICOS ligand; IL6, interleukin 6; LYN, tyrosine-protein kinase Lyn; MIA, melanoma-derived growth regulatory protein; MK, midkine; MUC16, mucin-16; NPX, normalized protein expression; *p*-val, nominal *p*-value; *p*-val^adj^, *p*-value obtained after adjustment for multiple testing by Benjamini–Hochberg; SCF, stem cell factor; TGFalpha, transforming growth factor alpha; TNFRSF4, tumor necrosis factor receptor superfamily member 4; TNFSF13B, tumor necrosis factor ligand superfamily member 13B; TRAIL, TNF-related apoptosis-inducing ligand; VEGFA, vascular endothelial growth factor A; VEGFR2, vascular endothelial growth factor receptor 2; VIM, vimentin; WFDC2, WAP four-disulfide core domain protein 2; 95% CI, 95% confidence interval.

## Data Availability

The analyzed datasets are not publicly available.
